# Editorial: New advances in prosthetic surgery of large joints

**DOI:** 10.3389/fsurg.2026.1789779

**Published:** 2026-03-05

**Authors:** Michela Saracco, Eugenio Jannelli

**Affiliations:** 1 Department of Orthopaedics, University of Naples “Federico II”, Naples, Italy; 2 Department of Orthopaedics, San Matteo Hospital Foundation (IRCCS), Pavia, Italy

**Keywords:** hip replacement, knee replacement, large joints replacements, orthopaedics, prosthetic surgery

It is our great pleasure to introduce this Research Topic on *New Advances in prosthetic surgery of large joints*. Osteoarthritis is a common cause of disability and chronic pain in patients over the age of 50. The knee, hip, and shoulder joints are the most affected.

Osteoarthritis (OA) is the most common arthritic disease and the leading cause of disability in the general population. It is characterized by synovial inflammation, osteophyte formation, and cartilage loss.

The pathophysiological basis of this disease is still under continuous investigation, focusing on the study of changes in synovial fluid composition, neoangiogenesis, and variations in chondrocyte behavior (1). At variance with other chronic inflammatory arthritis conditions, OA is characterized by severe cartilage damage and mild synovial inflammation due to two different molecular mechanisms: Toll-like receptors (TLR) activated by endogenous products and the activation of the complement cascade due to some components of the ECM. TLR activation induces different molecular pathways that stimulates chemokines and cytokines release, increase angiogenesis, and promote immune system activation.

The clinical presentation of the disease increases with age. Charnley, many years ago, proposed the first “modern” hip replacement model (2). Since then, numerous studies and research have refined surgical practice, the quality of implants, and their versatility, allowing today's replacement of any major joint in the body with excellent results.

Research into new technologies and techniques, however, remains ongoing and stimulating.

## Surgical approaches and outcomes evaluation

Traditional surgical approaches are gradually being complemented by new technologies to personalize the treatment and reduce intra- and post-operative complications. (Xu et al.) Pre-operative patient optimization, as well as the ability to perform complex procedures with local-regional anaesthesia, has allowed for shorter recovery times and a faster return to normal life. (Lee et al.) Likewise, optimized use of tourniquets (Zhang et al.) and bone cements (Chen et al.) has led to good, long-lasting clinical results and a lower incidence of complications, even in the immediate perioperative time, especially in patients with comorbidities. Furthermore, in selected patients with osteoarthritis, prosthetic replacement of both hips or knees, or of one knee and one hip, optimizes recovery times for the return to normal daily activities and sports, in some cases, without increasing the risk of perioperative complications. (Compagnoni et al.)

There is also growing interest in blood markers that can predict functional outcome after prosthetic replacement, such as uric acid. However, much remains to be understood in this field (Xia et al.).

## Prosthetic design and biomaterials

New high-performance prosthetic designs are launched on the market every year. But there is no doubt that traditional anatomical designs offer excellent clinical and radiographic results even decades after their first implantation. As is known, in patients affected by hip dysplasia, conic-shaped hip prosthetic models are still the first option in case of severe deformity. In these ones, in fact, Wagner's traditional femoral stem design still offers an excellent solution with more than encouraging long-term follow-up results (D'Angelo et al.).

Nevertheless, regarding hip prosthetics, short stems came onto the market about 20 years ago, demonstrating good medium- and long-term results with low failure rates. The goal is to obtain increasingly high-performance prosthetic implants, saving bone stock for possible future revisions (3).

The search for biomaterials is also constantly evolving. The goal is to find durable ones, aspiring to a 0% failure rate in the case of a properly positioned implant. Hard-on-hard bearings in hip prosthetics are undoubtedly the best option in this regard. Yet the only hard-on-hard one currently authorized on the market is a ceramic-on-ceramic bearing. The well-known ceramic-on-metal one was withdrawn from the market years ago because of causing harm to patient health due to the release of metal ions, chromium, and cobalt. However, several literature papers have shown that, in the case of correct positioning, wear is negligible and therefore the risk of metal ion release is minimal and not dangerous (4, 5). Ceramic-on-ceramic bearing is certainly the most reliable and long-lasting one, thanks to the development of Alumina and Zirconia-based composites. On the other hand, hard-on-hard bearings can be complicated by squeaking. Squeaking, even in the absence of pathological significance, can compromise the patient's quality of life. The causes are unknown, but the lubrication of the artificial joint is likely involved. Furthermore, in the case of ceramic-on-polyethylene bearings, poor lubrication can cause increased wear. Recent (bio)tribology studies have been conducted. These have led to the development of elasto-hydrodynamic lubrication models with promising results in *in-vitro* studies (6). Certainly, the most widely used bearing remains ceramic-on-polyethylene, which, however, has the disadvantage of having a higher polyethylene wear rate. Special treatments of the latter (vitamin E–enhanced polyethylene, crosslinking process, etc.) have, however, meant that this is much lower than in the past. Highly crosslinked polyethylene has significantly reduced wear rates in large prosthetic implants (7).

Special materials such as Oxidized Zirconium, on the other hand, find little application in hip replacement, but they are very common in knee prosthetics with good tolerability rates (8). Unfortunately, however, contact between the component and the metal surface leads to the development of rapid and catastrophic wear, which is why its use has been reduced (9).

## Prosthetic surgery in unusual pathological settings

Prosthetic replacements are often performed in patients with specific comorbidities, which can complicate the surgical procedure or be associated with higher complication rates. Performing surgical procedures on amputees is notoriously fraught with technical challenges, from patient positioning to the intraoperative maneuvers required for correct prosthetic placement. In this regard, the minimally invasive anterior hip approach represents a valid option, especially for patients with above-the-knee amputations (Zhao et al.)

Very rare diseases may also be associated with the risk of early osteoarthritis, such as Ehlers-Danlos syndrome. In these cases, it is essential to ensure well-positioned implants with low wear rates, given the patient's young age and the risk of future revisions (10, 11).

Similarly, the coexistence of systemic diseases that impact the joints often poses a challenge for the orthopedic surgeon. Hemophilia, in cases of recurrent hemarthrosis, especially of the knee, has highly destructive effects on joint structures. Indeed, prosthetic fitting is technically more complex, and complications are more frequent. It is therefore not surprising that more compromised preoperative situations, in terms of flexion contracture and reduced range of motion, correlate with worse postoperative outcomes. Pre-operative planning and patient optimization are mandatory in these cases (Jiang et al.).

## Modern technologies

The use of modern prosthetic technologies has allowed us to optimize the components' positioning and, therefore, the implant's duration over the years. New technologies acquire particular significance and importance, especially in the case of complex prosthetics, as occurs in post-traumatic outcomes, especially in elderly and osteoporotic patients (Liu et al.).

From guided positioning techniques, through 3D printing cutting masks, to robot-assisted prosthetics, the level of accuracy of prosthetic surgical procedures has continued to grow. Furthermore, 3D printing of the patient model obtained from CT scanning allows for extremely accurate planning of the procedure, as well as surgical simulation to optimize surgical steps and times (12). This, among other things, also helps reduce the risk of developing a peri-prosthetic joint infection. Additionally, accurate planning prevents the risk of implant instability, such as in the hip and shoulder.

## Perspectives and future directions

The future goal is to make this type of procedure as accessible as possible to patients with osteoarthritis by creating implants that are as personalized as possible. The use of new technologies and material improvements aims to reduce the rate of complications and prosthetic failures, ensuring high long-term success rates.

Many thanks to the authors and reviewers who contributed to this Research Topic in *Frontiers in Surgery, Orthopaedic Surgery*. We hope that it will help readers to get some insights into joint replacements and trigger further research in this field.
